# Assessing maternal postnatal depression, bonding and practices in mothers of preterm and low birth weight infants in Indonesia

**DOI:** 10.1016/j.mex.2025.103750

**Published:** 2025-12-05

**Authors:** Ricvan Dana Nindrea, Linda Rosalina, Milya Novera, Long Chiau Ming, Nissa Prima Sari, Nabil Aresto Avilla, Fanisha Anugrah Rahmadhani Putri, Nailah Putri Rivani

**Affiliations:** a Department of Medicine, Faculty of Medicine, Universitas Negeri Padang, Bukittinggi, Indonesia; b Department of Nursing, Faculty of Psychology and Health Sciences, Universitas Negeri Padang, Padang, Indonesia; c School of Medical and Life Sciences, Sunway University, Sunway City, Malaysia; d Maternal and Child Health Division, Solok District Health Office, Indonesia

**Keywords:** Postnatal depression, Mother-infant bonding, Practices, Preterm infants, Low birth weight, Indonesia

## Abstract

Preterm and low birth weight (LBW) infants face elevated health risks and require specialized care. Maternal postnatal depression (PND) and the quality of mother–infant bonding are critical determinants of caregiving practices and neonatal outcomes. However, practical, validated methods for assessing these constructs remain limited within the Indonesian clinical and research context. This study presents a protocol for assessing PND and bonding among mothers of preterm and LBW infants in Indonesia. A community-based cross-sectional design was implemented across three districts in West Sumatra. A total of 255 mothers of preterm or LBW infants were selected using multistage random sampling. PND was measured using the validated 10-item Edinburgh Postnatal Depression Scale (EPDS), with a cut-off score of 12/13 indicating significant depression. Mother–infant bonding was assessed with a culturally adapted 10-item bonding questionnaire. Maternal practices were evaluated using an 8-item checklist covering breastfeeding, Kangaroo Mother Care, immunization, and use of maternal–child health records. Instruments underwent expert review, translation and back-translation, and pilot testing to ensure validity and reliability (Cronbach’s α: 0.75–0.90). The primary endpoints included the identification of maternal PND, the quality of bonding, and maternal adherence to essential infant care practices. Data collection followed a standardized interviewer protocol, and data were analyzed using Partial Least Squares Structural Equation Modeling (PLS-SEM). The protocol proved feasible in community settings and provides a replicable method to evaluate maternal PND and bonding, with potential to inform interventions that enhance neonatal care outcomes.

## Graphical abstract

**Figure:**
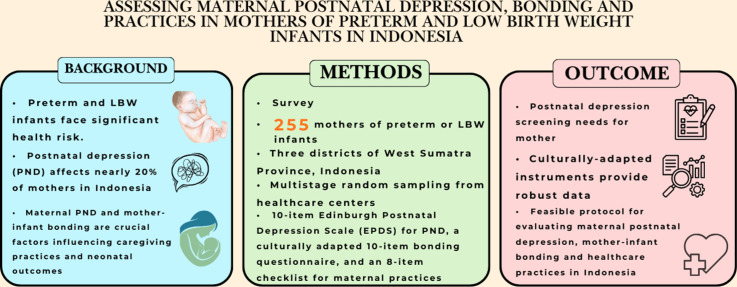

Specifications table

**Table:** 

**Subject area**	Medicine and Dentistry
**More specific subject area**	Maternal and child health; Neonatal care; Mental health
**Name of your protocol**	A protocol for assessing maternal PND, bonding and practices in mothers of preterm and LBW infants in Indonesia
**Experimental design**	A community-based cross-sectional study conducted from June to August 2025 across 3 districts in West Sumatra, Indonesia. A total of 255 mothers of preterm or low birth weight infants were selected using multistage random sampling. Data were collected using validated instruments (10-item EPDS for PND, a culturally adapted 10-item bonding questionnaire, and an 8-item checklist for maternal practices) administered by trained interviewers following a standardized protocol.
**Trial registration**	Not applicable
**Ethics**	Ethical approval was obtained from the Ethics Committee of Dr. M. Djamil Hospital, Padang, Indonesia (No. DP.04.03/D.XVI.XI/402/2025). Written informed consent was obtained from all participants prior to enrollment and data collection.
**Value of the Protocol**	• Integrates culturally adapted and validated instruments with strong psychometric properties • Enables evaluation of how maternal PND and bonding influence essential maternal practices for preterm and LBW infants. • Offers practical insights to inform maternal–child health interventions and policies in Indonesia.

## Background

Preterm and low birth weight (LBW) infants represent a major global health challenge, accounting for a substantial proportion of neonatal morbidity, mortality, and long-term developmental complications [1]. In Indonesia, the prevalence of preterm births is estimated at 10 % and LBW at 6 %, contributing significantly to stunting, recurrent infections, and neonatal deaths [2,3]. These infants often require specialized care, yet access to advanced neonatal services is uneven, particularly in rural and resource-constrained regions. As a result, maternal caregiving practices become essential in ensuring survival and healthy development.

Maternal postnatal depression (PND) is a critical determinant of caregiving capacity [4,5]. Affecting nearly 20 % of Indonesian mothers, PND not only compromises maternal well-being but also disrupts emotional responsiveness and the establishment of mother–infant bonding [6,7]. Impaired bonding has been associated with reduced adherence to recommended practices, such as exclusive breastfeeding, Kangaroo Mother Care, immunization compliance, and consistent use of maternal–child health records. These disruptions increase the vulnerability of preterm and LBW infants to preventable complications [[8], [9], [10]].

Despite its importance, maternal mental health remains under-recognized in Indonesia’s primary healthcare system. Screening for PND is rarely integrated into routine maternal and child health services, frontline health workers often lack training to detect depressive symptoms, and culturally adapted instruments are limited [[11], [12], [13]]. Furthermore, existing studies tend to emphasize clinical management of neonatal conditions, overlooking the psychosocial and behavioral dimensions of maternal care that are crucial in low- and middle-income contexts [14,15].

A systematic and culturally relevant protocol for assessing maternal PND, mother–infant bonding, and maternal practices is therefore urgently needed. Such a protocol would provide robust and replicable data to inform interventions, strengthen community-based maternal and neonatal services, and guide health policy aimed at improving outcomes for preterm and LBW infants. This study aims to develop and validate a protocol for assessing maternal PND and bonding among mothers of preterm and LBW infants in Indonesia, and to examine their influence on essential maternal practices in community settings.

## Description of protocol

This protocol was designed to assess maternal PND, bonding, and essential maternal practices among mothers of preterm and LBW infants in community settings in Indonesia.

### Study design and setting

This study employed a community-based cross-sectional design conducted between June and August 2025. The research took place in 3 districts of West Sumatra Province, Indonesia: Padang City, South Solok Regency, and Solok Regency. These sites were chosen to capture a diversity of maternal and child health contexts, ranging from urban to rural areas with varied access to healthcare facilities. Conducting the study across these regions allowed the protocol to be tested in settings that differ in socioeconomic and healthcare characteristics, thereby improving its generalizability and replicability in resource-limited contexts.

### Study population and sample size calculation

The study population consisted of mothers who had given birth to preterm infants (gestational age <37 weeks) or infants with LBW (<2500 g) between June and August 2025. The Edinburgh Postnatal Depression Scale (EPDS) is typically administered at 6–8 weeks postpartum, which is considered the optimal period for screening for postnatal depression [16]. A total sample of 255 mothers was calculated using a standard formula for proportions, assuming a 95 % CI, a hypothesized prevalence of 79 % for mothers with adequate bonding and support practices who adhered to recommended healthcare services for preterm and LBW infants (*p* = 0.79) [8], and a 5 % margin of error. Multistage random sampling was employed, health centers were first selected within each district, eligible mothers were listed, and participants were then chosen through randomization. Mothers were eligible if they provided informed consent and were able to communicate verbally. Those who were unavailable during the study period or declined participation were excluded.

### Measurement instruments

Maternal PND was assessed using the 10-item EPDS [16], which employs a four-point Likert scale ranging from 0 to 3. The scale was validated and culturally adapted to the Indonesian context. A threshold score of 12/13 on the EPDS was considered to indicate mothers likely suffering from postnatal depression of varying severity, requiring further clinical assessment. A score of 9/10 is recommended as an alternative threshold for routine use by primary care professionals. Participants who screened positively for self-harm (Item 10 of EPDS) were immediately referred to mental health professionals for further assessment and appropriate crisis management [9,16]. Mother–infant bonding was assessed using a 10-item bonding questionnaire, adapted from established scales, which utilizes a four-point Likert scale ranging from 0 to 3 [17,18], focusing on emotional closeness, responsiveness, and perceived quality of the maternal relationship with the infant. Essential maternal practices were assessed using an eight-item checklist evaluating maternal caregiving behaviors, with each item rated using a Yes/No response format [8,9], including Kangaroo Mother Care (KMC), exclusive breastfeeding, immunization adherence, and use of the Maternal and Child Health (MCH) book or Little Baby Handbook (LBH) for growth monitoring and record-keeping.

### Instrument adaptation and validation

All instruments underwent a rigorous adaptation process to ensure cultural and linguistic appropriateness. Each questionnaire was translated into Bahasa Indonesia and back-translated into English by independent bilingual experts. A panel of pediatricians, neonatologists, and psychologists reviewed the instruments to confirm content validity. Pilot testing was then conducted on 20 mothers outside the main study sites to evaluate clarity, comprehension, and acceptability. Based on participant feedback, minor revisions were made to improve readability. Reliability was confirmed with Cronbach’s alpha values ranging from 0.75 to 0.90, while confirmatory factor analysis demonstrated strong construct validity.

### Data collection procedure

Fieldworkers with qualifications in public health or nursing carried out the data collection after completing a two-day training program focused on research objectives, ethics, and questionnaire administration techniques. The training also included role-playing and mock interviews to enhance interview techniques and ensure standardization. Each interview lasted approximately 35 to 45 min and was conducted in participants’ homes or local health centers, depending on convenience and accessibility. Privacy and comfort were prioritized to encourage honest responses. Fieldworkers followed a standardized protocol for sequencing questions, probing, and recording responses. Supervisors conducted regular monitoring visits and daily debriefings to ensure consistency and adherence to the protocol across sites.

### Data management and analysis

Data were initially recorded on paper-based forms and then double-entered into SPSS version 25.0 to minimize input errors. Blinding of data entry was implemented to reduce bias. Double entry was used to detect discrepancies, and any inconsistencies were resolved through discussions between the data entry team and the research supervisor. Missing data were addressed using listwise deletion, excluding cases with incomplete responses, ensuring that only fully completed cases were included in the analysis.

Descriptive analyses were performed to summarize demographic and clinical characteristics using frequencies, percentages, and medians. For hypothesis testing, Partial Least Squares Structural Equation Modeling (PLS-SEM) was conducted with SmartPLS 4.0. PLS-SEM was preferred over covariance-based SEM (CB-SEM) because PLS-SEM is particularly well-suited for complex models with smaller sample sizes, where normality assumptions are difficult to meet. PLS-SEM maximizes variance explained and is more robust in situations with data that are not fully normally distributed, making it a more appropriate method for this study. The measurement model was evaluated for reliability using Cronbach’s alpha (α) (≥0.70 for acceptable internal consistency), Composite Reliability (CR) (≥0.70 for good reliability), and Average Variance Extracted (AVE) (≥0.50 for convergent validity). Outer loadings were required to exceed 0.70, and items with loadings below this threshold or those failing to meet discriminant validity (measured via the Fornell-Larcker criterion) were considered for removal. The structural model tested hypothesized pathways, with bootstrapping (5000 resamples) used to calculate t-values. Statistical significance was set at *p* < 0.05. Anticipated effect sizes for the structural paths ranged from 0.10 (small) to 0.30 (medium), consistent with similar PLS-SEM studies in health and behavioral sciences [[19], [20], [21]].

### Ethical considerations

Approval for the study was provided by the Ethics Committee of Dr. M. Djamil Hospital, Padang, Indonesia (ID: DP.04.03/D.XVI.XI/402/2025). Written informed consent was obtained from all participants prior to enrollment and data collection.

## Protocol validation

Maternal and birth characteristics of mothers with preterm and LBW infants (Table 1).

**Table 1:** Maternal and birth characteristics of mothers with preterm and LBW infants.

Variables	Value (*n* = 255)
Maternal age, median (Q1-Q3)	30 (17–42)
Number of prior pregnancies (including miscarriages and stillbirths), median (Q1-Q3)	2 (1–7)
Number of births (including stillbirths), median (Q1-Q3)	1 (0–5)
Gestational age at birth, f( %)	
Preterm	155 (63.0)
Full-term	91 (37.0)
Type of delivery, f( %)	
Vaginal delivery	107 (42.0)
Cesarean delivery	148 (58.0)
NICU admission, f( %)	
Yes	69 (27.1)
No	186 (72.9)
Delivery complications, f( %)	
No complications	172 (67.5)
Eclampsia	49 (19.2)
Postpartum hemorrhage	10 (3.9)
Other complications	24 (9.4)
Delivery attendant, f( %)	
Obstetric specialist	181 (71.0)
General practitioner	12 (4.7)
Midwife	57 (22.4)
Other attendants	5 (2.0)
Infant birth weight (g), median (Q1-Q3)	2200 (1300–2460)
Infant birth length (cm), median (Q1-Q3)	45 (37–49)
Maternal education, f( %)	
No formal schooling	24 (9.4)
Primary education	59 (23.1)
Junior high school education	103 (40.4)
Senior high school education	62 (24.3)
Higher education	7 (2.7)
Mother’s occupation, f( %)	
Housewife	184 (72.2)
Public servant	12 (4.7)
Self-employed	59 (23.1)

Table 1 presents the maternal and birth characteristics of 255 mothers with preterm and LBW infants. The median maternal age was 30 years (IQR: 17–42), with most mothers having at least junior high school education (68 %). The median number of prior pregnancies was 2 (IQR: 1–7), and the majority of deliveries were cesarean sections (58 %) due to the preterm nature of the infants, with 63 % of infants born preterm. The median birth weight was 2200 *g* (IQR: 1300–2460), and the median birth length was 45 cm (IQR: 37–49). Nearly 27 % of the infants required admission to the NICU, while the majority of births (71 %) were attended by obstetric specialists. Eclampsia (19.2 %) was the most common delivery complication, followed by postpartum hemorrhage (3.9 %).

Assessment of maternal PND, bonding and maternal practices factor loadings, reliability and validity (Table 2).

**Table 2:** Assessment of maternal PND, bonding and maternal practices factor loadings, reliability and validity.

Latent variables/ indicators	Standardized loadings
*Maternal PND (*α*=0.981; CR=0.984; AVE=0.857)*	
PD1 - Have you been able to find humor in situations?	0.970
PD2 - Do you look forward to things with enjoyment?	0.942
PD3 - Do you tend to blame yourself when things go wrong?	0.922
PD4 - Do you feel anxious or worried for no apparent reason?	0.896
PD5 - Do you experience feelings of panic without a clear cause?	0.872
PD6 - Do you feel like things are getting out of control?	0.904
PD7 - Have you had trouble sleeping due to unhappiness?	0.876
PD8 - Do you often feel sad or miserable?	0.938
PD9 - I Have you been so upset that you've cried?	0.981
PD10 - Have thoughts of harming yourself crossed your mind?	0.950
*Mother-infant bonding (*α*=0.986; CR=0.987; AVE=0.883)*	
MB1 - Do you feel a sense of love toward your baby?	0.911
MB2 - Do you feel fearful or panicked when caring for your baby?	0.953
MB3 - Do you ever feel hatred toward your baby?	0.963
MB4 - Do you feel emotionally disconnected from your baby?	0.915
MB5 - Do you experience anger toward your baby?	0.924
MB6- Do you enjoy spending time with your baby?	0.953
MB7 - Do you wish your baby were different?	0.930
MB8 - Do you feel protective over your baby?	0.961
MB9 - Do you wish you didn’t have your baby?	0.953
MB10 - Do you feel emotionally close to your baby?	0.934
*Maternal practices (*α*=0.946; CR=0.955; AVE=0.727)*	
MP1 - Do you perform KMC for your baby?	0.932
MP2 - Do you breastfeed your baby?	0.733
MP3 - Has your baby received their immunizations?	0.858
MP4 - Do you monitor your baby’s growth and development using the MCH book or LBH?	0.914
MP5 - Did you access neonatal health services for your baby during the first month?	0.931
MP6 - Do you use the MCH book to share information about your baby’s growth and care with family members?	0.762
MP7 - Is your last health service visit recorded in the MCH book or LBH?	0.842
MP8 - Do healthcare workers provide education when you visit health facilities?	0.825

Table 2 shows the reliability, validity, and factor loadings for maternal PND, mother-infant bonding, and maternal practices. For PND, the scale demonstrated excellent reliability with a Cronbach’s alpha of 0.981, CR of 0.984, and AVE of 0.857. All items had strong factor loadings (0.872–0.981). For mother-infant bonding, the reliability was also excellent (α = 0.986, CR = 0.987, AVE = 0.883), with factor loadings ranging from 0.911 to 0.963. For maternal practices, the reliability was good (α = 0.946, CR = 0.955, AVE = 0.727), with factor loadings between 0.733 and 0.931.

Analysis of discriminant validity for constructs (Table 3).

**Table 3:** Analysis of discriminant validity for constructs.

	Constructs	A	B	C
A	Maternal PND	*0.926*		
B	Mother infant bonding	0.140	*0.940*	
C	Maternal practices	0.198	0.164	*0.852*

Table 3 presents the results of discriminant validity analysis, where the diagonal values represent the AVE for each construct. The AVE values (italicized) show how much variance in each construct is explained by its indicators, with values above 0.50 generally indicating adequate convergent validity.

**Fig. 1:**
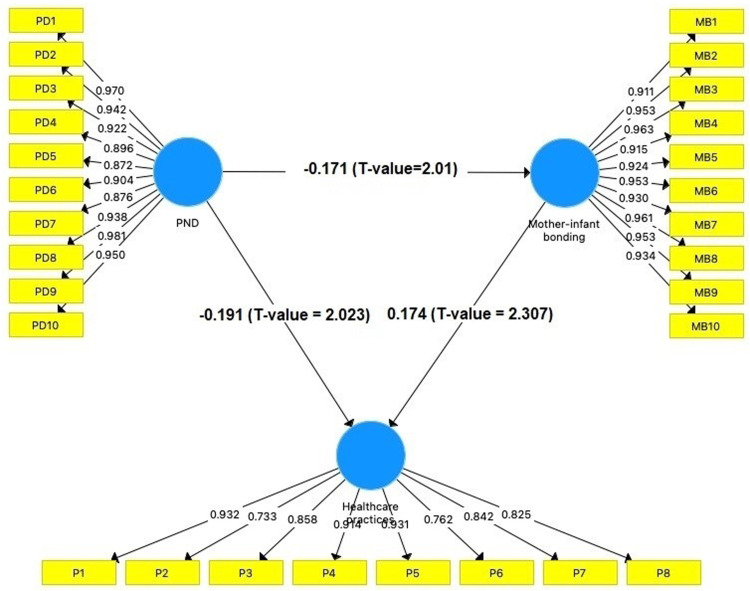
Structural model for maternal PND, bonding and maternal practices in mothers of preterm and LBW infants in Indonesia.

Path analysis outcomes for maternal PND, bonding and maternal practices in mothers of preterm and LBW infants in Indonesia (Table 4 and Fig. 1).

**Table 4:** Path analysis outcomes for maternal PND, bonding, and maternal practices in mothers of preterm and LBW infants in Indonesia.

Specified path	Coefficient (β)	T-value	P-value
Mother-infant bonding -> Maternal practices	0.174	2.307	0.021*
Maternal PND -> Maternal practices	−0.191	2.023	0.049*
Maternal PND -> Mother-infant bonding	−0.171	2.01	0.047*

⁎ *P* < 0.05, considered statistically significant.

Table 4 shows the results of the path analysis between maternal PND, bonding, and maternal practices in mothers of preterm and LBW infants in Indonesia. The analysis found that mother-infant bonding is linked to better maternal practices, with a coefficient of 0.174 (*p* = 0.021). Additionally, maternal postnatal depression negatively affects maternal practices (β = −0.191, *p* = 0.049). Finally, maternal depression is also negatively to bonding with the baby (β = −0.171, *p* = 0.047).

## Limitations

This study has several limitations. The use of a cross-sectional design limits the ability to establish causal relationships between maternal PND, bonding, and maternal practices; a longitudinal design would offer better insights into their long-term effects. Additionally, the study was conducted in only three districts in West Sumatra, which may not fully represent the broader population of mothers with preterm and LBW infants in Indonesia, thereby limiting its generalizability. The reliance on self-reported data also introduces the potential for response biases, and while the measurement instruments were adapted to the Indonesian context, cultural and linguistic nuances may not have been fully captured. Moreover, the study focused solely on maternal PND and did not account for other mental health conditions, such as anxiety or stress, which could also affect caregiving.

## Related research article

Nindrea RD, Ming LC, Sari NP. Maternal postnatal depression, bonding, and health care practices in providing essential services for preterm and low birth weight infants in Indonesia. Clin Epidemiol Glob Health. 2025;33:102,028. DOI: 10.1016/j.cegh.2025.102028.

## CRediT authorship contribution statement

**Ricvan Dana Nindrea:** Conceptualization, Methodology, Software, Investigation, Formal analysis, Writing – original draft. **Linda Rosalina:** Validation, Writing – review & editing. **Milya Novera:** Validation, Writing – review & editing. **Long Chiau Ming:** Validation, Writing – review & editing. **Nissa Prima Sari:** Validation, Writing – review & editing. **Nabil Aresto Avilla:** Investigation, Writing – review & editing. **Fanisha Anugrah Rahmadhani Putri:** Investigation, Writing – review & editing. **Nailah Putri Rivani:** Investigation, Writing – review & editing.

## Declaration of competing interest

The authors declare that they have no known competing financial interests or personal relationships that could have appeared to influence the work reported in this paper.

## Data Availability

Data will be made available on request.

## Appendix Supplementary materials

Image, application 1

Image, application 2

## References

[1] Saharoy R., Potdukhe A., Wanjari M., Taksande A.B. (2023). Postpartum depression and maternal care: exploring the complex effects on mothers and infants. Cureus.

[2] Dennis C.L. (2014). Psychosocial interventions for the treatment of perinatal depression. Best. Pr. Res. Clin. Obs. Gynaecol..

[3] Putri A.S., Wurisastuti T., Suryaputri I.Y., Mubasyiroh R. (2023). Postpartum depression in young mothers in urban and rural Indonesia. J. Prev. Med. Public. Health.

[4] Slomian J., Honvo G., Emonts P., Reginster J.Y., Bruy`ere O. (2019). Consequences of maternal postpartum depression: a systematic review of maternal and infant outcomes. Womens. Health. (L.).

[5] Tembo C., Portsmouth L., Burns S. (2023). Postnatal depression and its social-cultural influences among adolescent mothers: a cross sectional study. PLOS. Glob. Public. Health.

[6] Accortt E.E., Cheadle A.C., Dunkel Schetter C. (2015). Prenatal depression and adverse birth outcomes: an updated systematic review. Matern. Child. Health. J..

[7] Banerjee P.Nina, McFadden K., Shannon J.D., Davidson L.L. (2023). Preterm birth and other measures of infant biological vulnerability: associations with maternal sensitivity and infant cognitive development. Matern. Child. Health. J..

[8] Nindrea R.D., Ming L., Sari N.P. (2025). Social support, motivation, and maternal knowledge about providing essential health care services for preterm and low birth weight babies in West Sumatra Province, Indonesia. J. Health. Res..

[9] Nindrea R.D., Ming L.C., Sari N.P. (2025). Maternal postnatal depression, bonding, and health care practices in providing essential services for preterm and low birth weight infants in Indonesia. Clin. Epidemiol. Glob. Health.

[10] Netsi E., Pearson R.M., Murray L., Cooper P., Craske M.G., Stein A. (2018). Association of persistent and severe postnatal depression with child outcomes. JAMa. Psychiatry.

[11] Handa A., Gaidhane A., Choudhari S. (2024). Shedding light on maternal mental health in LMICs: a cornerstone of maternal and child health care. Discov. Ment. Health.

[12] Gureje O., Oladeji B.D., Montgomery A.A., Araya R., Bello T., Chisholm D. (2019). High- versus low-intensity interventions for perinatal depression delivered by non-specialist primary maternal care providers in Nigeria: cluster randomised controlled trial (the EXPONATE trial). Br. J. Psychiatry.

[13] Tse J.S.Y., Haslam N. (2021). Inclusiveness of the concept of mental disorder and differences in help-seeking between Asian and white Americans. Front. Psychol..

[14] Howard L.M., Khalifeh H. (2020). Perinatal mental health: a review of progress and challenges. World. Psychiatry.

[15] Dadi A.F., Miller E.R., Mwanri L. (2020). Postnatal depression and its association with adverse infant health outcomes in low- and middle-income countries: a systematic review and meta-analysis. BMC. Pregnancy. ChildBirth.

[16] Cox J.L., Holden J.M., Sagovsky R. (1987). Detection of postnatal depression. Development of the 10-item Edinburgh postnatal depression scale. Br. J. Psychiatry.

[17] Kumar R. (1997). Anybody’s child”: severe disorders of mother-to-infant bonding. Br. J. Psychiatry.

[18] Yoshida K., Yamashita H., Conroy S., Marks M., Kumar C. (2012). A Japanese version of mother-to-infant Bonding Scale: factor structure, longitudinal changes and links with maternal mood during the early postnatal period in Japanese mothers. Arch. Womens. Ment. Health.

[19] Ringle C.M., Sarstedt M., Sinkovics N., Sinkovics R.R. (2023). A perspective on using partial least squares structural equation modelling in data articles. Data. Br..

[20] Nazir M.F., Qureshi S.F. (2023). Applying structural equation modelling to understand the implementation of social distancing in the professional lives of healthcare workers. Int. J. Env. Res. Public. Health.

[21] Cruchinho P., López-Franco M.D., Capelas M.L., Almeida S., Bennett P.M., Miranda da Silva M. (2024). Translation, cross-cultural adaptation, and validation of measurement instruments: a practical guideline for novice researchers. J. Multidiscip. Heal..

