# Supplementary material for: Assessing maternal postnatal depression, bonding and practices in mothers of preterm and low birth weight infants in Indonesia
Source: MethodsX. 2025 Dec 5;16:103750. doi: 10.1016/j.mex.2025.103750 (PMC12743553; doi:10.1016/j.mex.2025.103750)
Supplement: Supplementary file 1 [file mmc1.docx]

**QUESTIONNAIRE**

**MATERNAL POSTNATAL DEPRESSION, BONDING AND PRACTICES IN MOTHERS OF PRETERM AND LOW BIRTH WEIGHT INFANTS IN INDONESIA**

Date: dd/mm/yyyy

**Section A. Maternal and birth characteristics**

| **No** | **Questions** | **Answer (Please tick)** |
| --- | --- | --- |
| A1 | Unique ID | [ ] [ ] [. ] [ ] |
| A2 | Maternal age | ______years |
| A3 | Number of prior pregnancies (including miscarriages and stillbirths) | ______ |
| A4 | Number of births (including stillbirths) | ______ |
| A5 | Gestational age at birth | [ ] 1. Preterm (<37 weeks)  [ ] 2. Full-term (≥37 weeks) |
| A6 | Type of delivery | [ ] 1. Vaginal delivery  [ ] 2. Cesarean delivery |
| A7 | NICU admission | [ ] 1. Yes  [ ] 2. No |
| A8 | Delivery complications | [ ] 1. No complications  [ ] 2. Eclampsia  [ ] 3. Postpartum hemorrhage  [ ] 4. Other (specify):_____ |
| A9 | Delivery attendant | [ ] 1. Obstetric specialist  [ ] 2. General practicioner  [ ] 3. Midwife  [ ] 4. Other (specify):_____ |
| A10 | Infant birth weight | ______grams |
| A11 | Infant birth length | ______cm |
| A12 | Maternal education level | [ ] 1. No formal schooling  [ ] 2. Primary education  [ ] 3. Junior high school  [ ] 4. Higher education (college/university) |
| A13 | Mother’s occupation | [ ] 1. Housewife  [ ] 2. Public servant  [ ] 3. Self-employed  [ ] 4. Other (specify):_____ |

**Section B. Maternal postnatal depression**

The Edinburg Postnatal Depression is a 10-item questionnaire. Women are asked to answer each question in terms of the past seven days.

| **No** | **Questions** | **Answer (Please tick)** |
| --- | --- | --- |
| B1 | Have you been able to find humor in situations? | [ ] 0. As much as I always could  [ ] 1. Not quite so much now  [ ] 2. Definitely not so much now  [ ] 3. Not at all |
| B2 | Do you look forward to things with enjoyment? | [ ] 0. As much as I always could  [ ] 1. Not quite so much now  [ ] 2. Definitely not so much now  [ ] 3. Not at all |
| B3 | Do you tend to blame yourself when things go wrong? | [ ] 0. No, never  [ ] 1. Not very often  [ ] 2. Yes, some of the time  [ ] 3. Yes, most of the time |
| B4 | Do you feel anxious or worried for no apparent reason? | [ ] 0. No, not at all  [ ] 1. Hardly ever  [ ] 2. Yes, sometimes  [ ] 3. Yes, very often |
| B5 | Do you experience feelings of panic without a clear cause? | [ ] 0. No, not at all  [ ] 1. No, not much  [ ] 2. Yes, sometimes  [ ] 3. Yes, quite a lot |
| B6 | Do you feel like things are getting out of control? | [ ] 0. No, I have been coping as well as ever  [ ] 1. No, most of the time I have coped quite well  [ ] 2. Yes, sometimes I haven’t been coping as well as usual  [ ] 3. Yes, most of the time I haven’t been able to cope at all |
| B7 | Have you had trouble sleeping due to unhappiness? | [ ] 0. No, not at all  [ ] 1. Not very often  [ ] 2. Yes, sometimes  [ ] 3. Yes, most of the time |
| B8 | Do you often feel sad or miserable? | [ ] 0. No, not at all  [ ] 1. Not very often  [ ] 2. Yes, quite often  [ ] 3. Yes, most of the time |
| B9 | I Have you been so upset that you've cried? | [ ] 0. No, never  [ ] 1. Only occasionally  [ ] 2. Yes, quite often  [ ] 3. Yes, most of the time |
| B10 | Have thoughts of harming yourself crossed your mind? | [ ] 0. Never  [ ] 1. Hardly ever  [ ] 2. Sometimes  [ ] 3. Yes, quite often |

**Section C. Mother-infant bonding**

We would like to understand how you have been feeling toward your baby recently. Below are some statements describing common feelings mothers may experience about their babies. Please select the option that best represents how you usually feel about your baby, rather than how you feel just today.

| **No** | **Questions** | **Answer (Please tick)** |
| --- | --- | --- |
| C1 | Do you feel a sense of love toward your baby? | [ ] 0. Not at all  [ ] 1. Slightly, some of the time  [ ] 2. Very much so, some of the time  [ ] 3. Very much so, most of the time |
| C2 | Do you feel fearful or panicked when caring for your baby? | [ ] 0. Very much so, most of the time  [ ] 1. Very much so, some of the time  [ ] 2. Slightly, some of the time  [ ] 3. Not at all |
| C3 | Do you ever feel hatred toward your baby? | [ ] 0. Very much so, most of the time  [ ] 1. Very much so, some of the time  [ ] 2. Slightly, some of the time  [ ] 3. Not at all |
| C4 | Do you feel emotionally disconnected from your baby? | [ ] 0. Very much so, most of the time  [ ] 1. Very much so, some of the time  [ ] 2. Slightly, some of the time  [ ] 3. Not at all |
| C5 | Do you experience anger toward your baby? | [ ] 0. Very much so, most of the time  [ ] 1. Very much so, some of the time  [ ] 2. Slightly, some of the time  [ ] 3. Not at all |
| C6 | Do you enjoy spending time with your baby? | [ ] 0. Not at all  [ ] 1. Slightly, some of the time  [ ] 2. Very much so, some of the time  [ ] 3. Very much so, most of the time |
| C7 | Do you wish your baby were different? | [ ] 0. Very much so, most of the time  [ ] 1. Very much so, some of the time  [ ] 2. Slightly, some of the time  [ ] 3. Not at all |
| C8 | Do you feel protective over your baby? | [ ] 0. Not at all  [ ] 1. Slightly, some of the time  [ ] 2. Very much so, some of the time  [ ] 3. Very much so, most of the time |
| C9 | Do you wish you didn’t have your baby? | [ ] 0. Very much so, most of the time  [ ] 1. Very much so, some of the time  [ ] 2. Slightly, some of the time  [ ] 3. Not at all |
| C10 | Do you feel emotionally close to your baby? | [ ] 0. Not at all  [ ] 1. Slightly, some of the time  [ ] 2. Very much so, some of the time  [ ] 3. Very much so, most of the time |

**Section D. Maternal practices**

| **No** | **Questions** | **Answer (Please tick)** |
| --- | --- | --- |
| D1 | Do you perform KMC for your baby? | [ ] 0. No  [ ] 1. Yes |
| D2 | Do you breastfeed your baby? | [ ] 0. No  [ ] 1. Yes |
| D3 | Has your baby received their immunizations? | [ ] 0. No  [ ] 1. Yes |
| D4 | Do you monitor your baby’s growth and development using the MCH book or LBH? | [ ] 0. No  [ ] 1. Yes |
| D5 | Did you access neonatal health services for your baby during the first month? | [ ] 0. No  [ ] 1. Yes |
| D6 | Do you use the MCH book to share information about your baby’s growth and care with family members? | [ ] 0. No  [ ] 1. Yes |
| D7 | Is your last health service visit recorded in the MCH book or LBH? | [ ] 0. No  [ ] 1. Yes |
| D8 | Do healthcare workers provide education when you visit health facilities? | [ ] 0. No  [ ] 1. Yes |
