# Supplementary material for: Assessing maternal postnatal depression, bonding and practices in mothers of preterm and low birth weight infants in Indonesia
Source: MethodsX. 2025 Dec 5;16:103750. doi: 10.1016/j.mex.2025.103750 (PMC12743553; doi:10.1016/j.mex.2025.103750)
Supplement: Supplementary file 2 [file mmc2.docx]

**KUESIONER**

**DEPRESI PASCA PERSALINAN, IKATAN IBU-ANAK, DAN PRAKTIK IBU PADA BAYI PREMATUR DAN BERAT BADAN LAHIR RENDAH DI INDONESIA**

Tanggal: __/__/____

**Bagian A. Karakteristik ibu dan persalinan**

| **No** | **Pertanyaan** | **Jawaban (Beri tanda** ✓) |
| --- | --- | --- |
| A1 | ID unik | [ ] [ ] [. ] [ ] |
| A2 | Usia ibu | ______tahun |
| A3 | Jumlah kehamilan sebelumnya (termasuk keguguran dan lahir mati) | ______ |
| A4 | Jumlah persalinan (termasuk lahir mati) | ______ |
| A5 | Usia kehamilan saat melahirkan | [ ] 1. Prematur (<37 minggu)  [ ] 2. Aterm (≥37 minggu) |
| A6 | Jenis persalinan | [ ] 1. Persalinan normal  [ ] 2. Operasi sesar |
| A7 | Bayi dirawat di NICU | [ ] 1. Ya  [ ] 2. Tidak |
| A8 | Komplikasi persalinan | [ ] 1. Tidak ada komplikasi  [ ] 2. Eklampsia  [ ] 3. Perdarahan pascapersalinan  [ ] 4. Lainnya (sebutkan):_____ |
| A9 | Penolong persalinan | [ ] 1. Dokter spesialis obstetri  [ ] 2. Dokter umum  [ ] 3. Bidan  [ ] 4. Lainnya (sebutkan):_____ |
| A10 | Berat lahir bayi | ______grams |
| A11 | Panjang badan bayi saat lahir | ______cm |
| A12 | Pendidikan terakhir ibu | [ ] 1. Tidak sekolah  [ ] 2. Sekolah dasar  [ ] 3. Sekolah menengah pertama  [ ] 4. Sekolah menengah atas  [ ] 5. Pendidikan tinggi (perguruan tinggi/ universitas) |
| A13 | Pekerjaan ibu | [ ] 1. Ibu rumah tangga  [ ] 2. Pegawai negeri/ swasta  [ ] 3. Wiraswasta  [ ] 4. Lainnya (sebutkan):_____ |

**Bagian B. Depresi pasca persalinan**

Kuesioner ini terdiri dari 10 pertanyaan. Ibu diminta menjawab berdasarkan perasaan selama 7 hari terakhir.

| **No** | **Pertanyaan** | **Jawaban (Beri tanda** ✓) |
| --- | --- | --- |
| B1 | Apakah Anda masih dapat menemukan hal-hal lucu dalam berbagai situasi? | [ ] 0. Sama seperti biasanya  [ ] 1. Tidak sebanyak biasanya  [ ] 2. Jelas tidak sebanyak biasanya  [ ] 3. Sama sekali tidak |
| B2 | Apakah Anda menantikan sesuatu dengan perasaan senang? | [ ] 0. Sama seperti biasanya  [ ] 1. Tidak sebanyak biasanya  [ ] 2. Jelas tidak sebanyak biasanya  [ ] 3. Sama sekali tidak |
| B3 | Apakah Anda cenderung menyalahkan diri sendiri ketika sesuatu berjalan tidak baik? | [ ] 0. Tidak, tidak pernah  [ ] 1. Tidak terlalu sering  [ ] 2. Ya, kadang-kadang  [ ] 3. Ya, sebagian besar waktu |
| B4 | Apakah Anda merasa cemas atau khawatir tanpa alasan yang jelas? | [ ] 0. Tidak, sama sekali tidak  [ ] 1. Hampir tidak pernah  [ ] 2. Ya, kadang-kadang  [ ] 3. Ya, sangat sering |
| B5 | Apakah Anda pernah merasa panik tanpa penyebab yang jelas? | [ ] 0. Tidak, sama sekali tidak  [ ] 1. Tidak, tidak terlalu sering  [ ] 2. Ya, kadang-kadang  [ ] 3. Ya, cukup sering |
| B6 | Apakah Anda merasa seolah-olah keadaan di luar kendali Anda? | [ ] 0. Tidak, saya mengatasinya sama baiknya seperti biasanya  [ ] 1. Tidak, sebagian besar waktu saya dapat mengatasinya dengan baik  [ ] 2. Ya, kadang-kadang saya tidak dapat mengatasinya sebaik biasanya  [ ] 3. Ya, sebagian besar waktu saya sama sekali tidak dapat mengatasinya |
| B7 | Apakah Anda kesulitan tidur karena merasa tidak bahagia? | \| [ ] 0. Tidak, sama sekali tidak  [ ] 1. Tidak terlalu sering  [ ] 2. Ya, kadang-kadang  [ ] 3. Ya, sebagian besar waktu \| \| --- \|  \|  \| \| --- \| |
| B8 | Apakah Anda sering merasa sedih atau sengsara? | [ ] 0. Tidak, sama sekali tidak  [ ] 1. Tidak terlalu sering  [ ] 2. Ya, cukup sering  [ ] 3. Ya, sebagian besar waktu |
| B9 | Apakah Anda merasa sangat sedih hingga menangis? | [ ] 0. Tidak, tidak pernah  [ ] 1. Hanya sesekali  [ ] 2. Ya, cukup sering  [ ] 3. Ya, sebagian besar waktu |
| B10 | Apakah Anda pernah terpikir untuk menyakiti diri sendiri? | [ ] 0. Tidak pernah  [ ] 1. Hampir tidak pernah  [ ] 2. Kadang-kadang  [ ] 3. Ya, cukup sering |

**Bagian C. Ikatan ibu-bayi**

Kami ingin memahami bagaimana perasaan Anda terhadap bayi Anda akhir-akhir ini. Di bawah ini terdapat beberapa pernyataan yang menggambarkan perasaan umum para ibu terhadap bayinya. Silakan pilih jawaban yang paling menggambarkan bagaimana Anda biasanya merasa terhadap bayi Anda, bukan hanya hari ini.

| **No** | **Pertanyaan** | **Jawaban (Beri tanda** ✓) |
| --- | --- | --- |
| C1 | Apakah Anda merasa memiliki kasih sayang terhadap bayi Anda? | [ ] 0. Tidak sama sekali  [ ] 1. Sedikit, kadang-kadang  [ ] 2. Sangat banyak, kadang-kadang  [ ] 3. Sangat banyak, sebagian besar waktu |
| C2 | Apakah Anda merasa takut atau panik saat merawat bayi Anda? | [ ] 0. Sangat banyak, sebagian besar waktu  [ ] 1. Sangat banyak, kadang-kadang  [ ] 2. Sedikit, kadang-kadang  [ ] 3. Tidak sama sekali |
| C3 | Apakah Anda pernah merasa benci terhadap bayi Anda? | [ ] 0. Sangat banyak, sebagian besar waktu  [ ] 1. Sangat banyak, kadang-kadang  [ ] 2. Sedikit, kadang-kadang  [ ] 3. Tidak sama sekali |
| C4 | Apakah Anda merasa tidak memiliki kedekatan emosional dengan bayi Anda? | [ ] 0. Sangat banyak, sebagian besar waktu  [ ] 1. Sangat banyak, kadang-kadang  [ ] 2. Sedikit, kadang-kadang  [ ] 3. Tidak sama sekali |
| C5 | Apakah Anda pernah merasa marah terhadap bayi Anda? | [ ] 0. Sangat banyak, sebagian besar waktu  [ ] 1. Sangat banyak, kadang-kadang  [ ] 2. Sedikit, kadang-kadang  [ ] 3. Tidak sama sekali |
| C6 | Apakah Anda menikmati waktu yang dihabiskan bersama bayi Anda? | [ ] 0. Tidak sama sekali  [ ] 1. Sedikit, kadang-kadang  [ ] 2. Sangat banyak, kadang-kadang  [ ] 3. Sangat banyak, sebagian besar waktu |
| C7 | Apakah Anda berharap bayi Anda berbeda dari yang sekarang? | [ ] 0. Sangat banyak, sebagian besar waktu  [ ] 1. Sangat banyak, kadang-kadang  [ ] 2. Sedikit, kadang-kadang  [ ] 3. Tidak sama sekali |
| C8 | Apakah Anda merasa ingin melindungi bayi Anda? | [ ] 0. Tidak sama sekali  [ ] 1. Sedikit, kadang-kadang  [ ] 2. Sangat banyak, kadang-kadang  [ ] 3. Sangat banyak, sebagian besar waktu |
| C9 | Apakah Anda pernah berharap tidak memiliki bayi ini? | [ ] 0. Sangat banyak, sebagian besar waktu  [ ] 1. Sangat banyak, kadang-kadang  [ ] 2. Sedikit, kadang-kadang  [ ] 3. Tidak sama sekali |
| C10 | Apakah Anda merasa memiliki kedekatan emosional dengan bayi Anda? | [ ] 0. Tidak sama sekali  [ ] 1. Sedikit, kadang-kadang  [ ] 2. Sangat banyak, kadang-kadang  [ ] 3. Sangat banyak, sebagian besar waktu |

**Bagian D. Praktik ibu**

| **No** | **Pertanyaan** | **Jawaban (Beri tanda** ✓) |
| --- | --- | --- |
| D1 | Apakah Anda melakukan Perawatan Metode Kanguru (KMC) untuk bayi Anda? | [ ] 0. Tidak  [ ] 1. Ya |
| D2 | Apakah Anda menyusui bayi Anda? | [ ] 0. Tidak  [ ] 1. Ya |
| D3 | Apakah bayi Anda telah menerima imunisasi? | [ ] 0. Tidak  [ ] 1. Ya |
| D4 | Apakah Anda memantau pertumbuhan dan perkembangan bayi menggunakan Buku KIA atau Buku Bayi Kecil? | [ ] 0. Tidak  [ ] 1. Ya |
| D5 | Apakah Anda mengakses layanan kesehatan neonatal untuk bayi Anda pada bulan pertama setelah lahir? | [ ] 0. Tidak  [ ] 1. Ya |
| D6 | Apakah Anda menggunakan Buku KIA untuk berbagi informasi mengenai pertumbuhan dan perawatan bayi kepada anggota keluarga? | [ ] 0. Tidak  [ ] 1. Ya |
| D7 | Apakah kunjungan layanan kesehatan terakhir Anda tercatat dalam Buku KIA atau Buku Bayi Kecil? | [ ] 0. Tidak  [ ] 1. Ya |
| D8 | Apakah tenaga kesehatan memberikan edukasi atau konseling saat Anda berkunjung ke fasilitas kesehatan? | [ ] 0. Tidak  [ ] 1. Ya |
